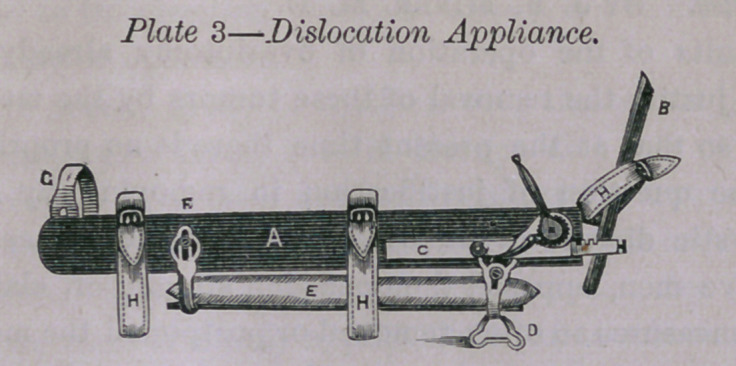# Lake Erie Medical Society

**Published:** 1869-06

**Authors:** 


ART. II.—*Lake Erie Medical Society. Abstract of Proceedings of a Meeting held at Dunkirk, May 12th and 13th*, 1869.

The Association met pursuant to order, at the reception rooms of the Erie Hotel, and was called to order by the President, Dr. D. D. Loop of North East, Pa.

The minutes of the semi-annual meeting held in Erie, October, 1868, were read and approved.

The officers elected for the ensuing year were—Drs. Edson Boyd of Ashville, N. Y., President; S. M. Smith of Dunkirk, Vice President; Charles Hazeltine of Jamestown, Secretary and Treasurer; Drs. Wallace of Erie, Pa., Strong of Westfield, and G. W. Hazeltine of Jamestown, Executive Committee.

Dr. Dean of Portland, reported a case of eruptive disease, simulating variola, then under treatment, in which the diagnosis was uncertain, and from the presence of variola in an adjoining town much excitement prevailed in the community where the case occurred. The subject was discussed by Drs. Dean, Strong of Westfield, G. W. Hazeltine of Jamestown, and Benedict, Rogers and Smith of Dunkirk, and the opinion was expressed that it was a severe case of varicella.

Dr. Chase of Mayville, reported a case of variola, in which purpura hæmorragica shew itself early in the disease, and it became rapidly fatal.

Drs. Rogers and Van Peyma presented a case of complete dislocation of the tibia and fibula, attended with caries of the tibia at intervals along its spine to the malleolus, with solution of continuity two inches below the tibio-femoral articulation. The subject was a German lad of twelve years, of apparently good constitution and one of a large family of healthy, robust children. The cause for this disease was from thumping the toe of a new and tightly-fitting boot in efforts to get it upon the foot. Pain and tumefaction immediately followed, and the case was placed in the care of one not a regular practitioner. It was treated at intervals for seven months by different irregular pathists with the supposition that it was a case of sprain, rheumatism, fever sores, etc. Drs. Rogers and Van Peyma visited the case, (several miles in the country,) five weeks since, and found the patient in his present condition, with the exception that the solution of continuity was not then complete. The shortening was two and one-half inches. There was no constitutional disturbance, the appetite was good, and he was able to move about the house with the aid of his hands and the other leg. The parents objecting to any severe operation being performed for the benefit of the boy, they were left with the suggestion that they bring him before this Society for examination. The point of interest in the case arises from the slight cause which produced so serious results. The case commencing in a slight subluxation might have misled those of higher pretensions and larger experience. It was discussed by Drs. Rogers, Van Peyma, Benedict and Smith of Dunkirk, Hazeltine of Jamestown, Strong of Westfield, Thompson of Stockton, etc. The conclusion arrived at was, that the leg should be removed.

Dr. Loop reported a case of fracture of the frontal bone occurring in North East, Pa. The patient, riding along the streets was rapidly approached from behind by a runaway team, and in the collision which followed, received a blow in the forehead, which fractured the bone to the extent of two inches in length by one in width, finely comminuting it, and deeply penetrating the cerebral substance. The man stopped his horses and secured them without assistance. Blood and brains flowed freely down his face, and a portion of brain protruded through the opening. Drs. Loop and Griffin dressed the wound, and report that nearly one hundred pieces of bone were removed, varying in size, from pins' heads to that of a large pea, some being embedded more than an inch in the substance of the brain. The day following the accident he was able to go to his home, two miles distant, and no untoward symptom followed this wound from its reception until its recovery, no coma, delirium, or severe pain, and but slight fever, and it healed kindly.

Adjourned to meet at 9 o'clock A. M.

*Thursday morning.*—President Loop and Vice President Thompson being absent, Dr. Smith was called to the chair.

Drs. Williams, Hazeltine and Spencer, were appointed a committee to nominate delegates to represent the Society at the next annual meeting of the American Medical Association, to be held in Washington.

Drs. H. R. Rogers of Dunkirk, G. W. Hazeltine and —— Rathburn of Jamestown, John Spencer of Westfield, L. G. Hall of Wattsburg, Pa., and —— Thayer of Erie, Pa., were nominated and confirmed.

Drs. Strong, Stubbs, Chase, Dean, Spencer and Moore, were elected as delegates to attend the meeting of the Pennsylvania State Medical Society to be held in Erie in June.

Dr. Rogers presented appliances for adjusting and treating fractures and for reducing dislocations, the application of which he demonstrated before the Society upon a gentleman present. The full set presented were calculated to meet the indications for treatment in most cases of fracture of thigh, leg, arm, fore-arm and clavicle, and the various dislocations of shoulder, elbow, wrist, hip, knee and ankle. The accompanying plates, with descriptions, will convey an imperfect idea of a portion of the appliances:

*Plate No.* 1.—For treating fractures of the femur, with or without extension and counter-extension.

A A—Long splint in two parts, united by joint and secured by ferrule (B.)

C—Footpiece attached to extension bar (E.)

D—Extending instrument with ratchet, catch, extension bar and lever (F.)

G—Foot and leg support and rest with bran cushion (H.)

I—Thigh rest, secured to side splint by K K, and fastened by thumb-screws, and made adjustable to suit a thin or muscular thigh and to adapt to the form of the thigh.

L L L—Straps furnished with buckles.

M—Aperture for counter-extending cords.

N N—Windlass shaped instruments with spring, catch and lever, with hole passing through shaft of each.

These latter are used as follows: Supplementary, narrow splints, are placed on the top and inner side of the thigh, and strong cords are carried through the instruments and around the limb and the ends tied tightly together. The lever tightens the cords to any desirable extent. This arrangement supplies the place of bandages, and when in use prevents the necessity of keeping up the extension and counter-extension.

As applied by Dr. R., counter-extension is effected in the early stage of the treatment, when strong muscular contraction is to be overcome, by the perineal roll, fastened at the counter-extending aperture. The perineum to be protected by soft compresses. Following or alternating with the roll, broad adhesive straps should be applied to inner and outer portions of the thigh, and secured by straps crossing these and alternating by bandage. These should be applied at the first dressing of the limb and save the necessity of disturbing the patient when their use becomes desirable. The ends of the plasters should be twisted into the form of cords for convenience of tying. The extending appliances should be adhesive straps secured by bandage. The straps, also twisted into the form of cords and carried through apertures in the extending bar on either side of the foot-piece and tied together. Extension is then made by turning the lever F until the ends of the fractured bone are in apposition. The limb is then secured to the side splint by means of the straps. This splint, as also the following, may be used for either right or left limb:

*Plate 2—Leg Splint.*

A Splint, B Foot-piece, C Extending Instrument, D E F Supports for foot and leg and long bran cushion; the cushion to be moulded to the form of lower part of the leg; G Counter-extending arch, with apertures at either extremity through which the counter-extending cords pass to be tied in the groove at its upper surface; H H H Straps.

Counter-extension is effected by adhesive straps applied on each side of the leg and knee, and secured by straps crossing these or by bandage. The ends twisted as before mentioned, and passed through the apertures and tied in the groove; extension as before described.

*Plate 3—Dislocation Appliance.*

A Splint, B Extension, C Counter-extending bar, D Lever, E Crutch head.

In dislocations of humerus and femur firm pads are applied above the condyles and secured by looped bands fastened by straps and buckles. Cords pass through the loops and around the extending bar. When sufficient extension is effected the surgeon manipulates the bone and an assistant presses upon the catch which causes the strain to yield quickly.

To enumerate all the appliances and their uses would require too much of space. We need only mention farther that the little objects described in plate 1 as windlass shaped, are applied to narrow splints in the treatment of fractures of the femur without extension and counter-extension, and to fractures of arm and forearm.

Drs. Strong, Hazeltine and Hall, were chosen a committee to revise the fee bill of the Society, and a report was presented, amended and adopted, and five hundred copies of the amended bill and Constitution ordered to be printed.

Subjects to be discussed at next meeting—Bright's Disease, Influenza as observed the past winter, and Extension and Counter-extension in fractures.

On motion of Dr. G. W. Hazeltine, Drs. Rogers and Van Peyma were chosen a committee to report the proceedings of this meeting for publication in the *Buffalo Medical and Surgical Journal.*

Adjourned.

H. R. ROGERS,
W. VAN PEYMA.
Committee.

## Figures and Tables

**Plate No. 1. f1:**
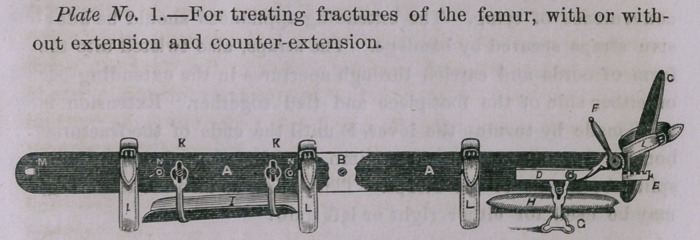


**Plate 2 f2:**
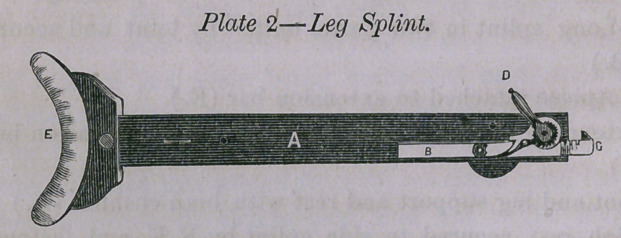


**Plate 3 f3:**