# Correction—Misplaced Cuts

**Published:** 1869-06

**Authors:** 


Correction—Misplaced Cuts.—It will be observed in the proceedings of the Lake Erie Medical Society, that the cuts which represent Dr. H. R. Rogers' surgical splints are misplaced, the leg splint occupying the place designed as described as the dislocation appliance. This is one of the accidents which human invention and ingenuity cannot provide against, and must be accepted as one of the penalties of Adam's transgression. Our readers will correct the mistake for themselves, as it is now too late for us to remedy it. We regret it, especially since the splints, when properly understood, are eminently suited to the objects in view, and worthy the consideration of all surgeons.

